# Practical Points in Diagnosis and Treatment

**Published:** 1915-04-10

**Authors:** 


### PRACTICAL POINTS IN DIAGNOSIS AND TREATMENT.

**Opium.**

Some patients who cannot take a dose of morphine by itself without being nauseated will take it combined with the aromatic spirit of ammonia without any difficulty.—Dr. Burney Yeo.

**Rum and Milk.**

Dr. Sidney H. Hall regards rum and milk as a valuable nourishing stimulant and fat producer. He advises the old Jamaica spirit; a tablespoonful to half a tumblerful of milk (sweetened if preferred) half an hour before breakfast as an appetiser for phthisical patients. He says also that many patients who cannot take milk in any other fashion take it readily on the addition of rum in the above proportions.—*Practitioner.*

**To Check a Common Cold.**

The following draught should be given at bed-time at the outset of the attack: Morphine hydrochloride, $\frac{1}{4}$ grain; spirit of nitrous ether, ʒj; solution of ammonium acetate, ʒiv; camphor water to ℥iss. After expelling mucus from the nostrils, these should be thoroughly sprayed every hour with a solution of menthol and eucalyptus in paroleine.

**Lithæmic Vertigo.**

Murchison applied this name to a condition in which vertigo comes on suddenly and is so severe that the patient is obliged to remain in the recumbent position and to keep perfectly still for hours or days, any attempt to raise the head causing renewed giddiness. In some of these cases mercury is successful, in other podophyllin.

**Intra-thoracic Tumours.**

In the differential diagnosis between an intra-thoracic aneurism and a solid tumour it may be helpful to remember that with a neoplasm an irregular pyrexia is not uncommon, while temperature disturbances in aneurism are extremely rare.

**Iodoform in Phthisis.**

A dose of one to two grains of iodoform can be prescribed over long periods if combined with codeine. It tends to check cough, to increase weight, and to improve the appetite.

**Menière's Disease.**

Babinski has advocated lumbar puncture in this disease. In some instances two punctures at intervals of a fortnight or so appear to have given prolonged or even permanent relief.

**Asthma.**

A combination of $\frac{1}{8}$ grain of morphine with 5 to 10 minims of solution of adrenalin (1 in 1,000) is often efficient in checking the asthmatic paroxysm. This, of course, does not apply to the so-called uræmic asthma, in which opium preparations are contra-indicated.

**A Mouth Wash.**

The following is a useful formula: Acid. benzoic., grs. xviij; tinct. eucalypti, ʒiss; S.V.R., ℥iss; ol. cinnam. (*vel.* ol. menth. pip.), ♏iij. Add a teaspoonful to half a glass of water.

**Interstitial Keratitis.**

This, as well as other evidences of inherited syphilis, may be postponed until adult life, certainly as late as the thirties; and while both eyes are usually involved, they are not always attacked at the same time; indeed the interval between the attacks may be a matter of years.

**Chloral Hydrate.**

The statement, frequently made, that chloral hydrate depresses the heart is of very questionable value. It has led to much timid prescribing and to the neglect of an excellent hypnotic.

**Leukæmia.**

The diagnosis in cases of leukæmia is sometimes missed because there is no enlargement of the spleen, and any enlargement of the superficial lymphatic glands is slight. Hence the possibility of the disease does not occur to the practitioner, and so the blood is not examined. The safe rule in any obscure case is to examine the lymphatic system, just as other parts of the body are examined. Even though the result seems negative, the blood should be tested, for the affected glands may be not those of the surface, but deeply situated and out of the reach of palpation.

**Hæmorrhoids.**

Avoid constipation and encourage moderate walking exercise. Wash with soap and water two or three times daily. If this proves insufficient, after each washing bathe freely with 1 in 1,000 perchloride of mercury. Gently reduce the hæmorrhoids and let the patient rest in the recumbent position for, say, half an hour. This treatment will often avoid operation.

**Peripheral Neuritis.**

A neuritis manifested by weakness and muscular atrophy and loss of tendon jerks may be associated with glycosuria, even though the patient has no symptoms suggestive of this latter condition. Unless the urine is tested the true nature of the case may therefore be overlooked.

**Empyema.**

The absence of any temperature disturbance is no proof that a pleural effusion is not purulent. This is especially true in the case of children, but it applies, at least occasionally, to adults also.

**Dyspepsia.**

In cases of severe indigestion in young women a period of complete rest in bed is often essential. Dr. F. J. Smith advises that no food be allowed after 5 p.m., and that a dram of bismuth be given at 10 p.m., 2 a.m., and 6 a.m.—that is, on a perfectly empty stomach.

**Migraine.**

When the attacks are frequent, a mixture containing 15 grains of the mixed bromides with a minim of solution of nitro-glycerine thrice daily and continued for several weeks is often very useful.

